# A pan-African spatial assessment of human conflicts with lions and elephants

**DOI:** 10.1038/s41467-021-23283-w

**Published:** 2021-05-20

**Authors:** Enrico Di Minin, Rob Slotow, Christoph Fink, Hans Bauer, Craig Packer

**Affiliations:** 1 Helsinki Lab of Interdisciplinary Conservation Science, Department of Geosciences and Geography, University of Helsinki, Helsinki, Finland; 2 Helsinki Institute of Sustainability Science (HELSUS), University of Helsinki, Helsinki, Finland; 3 School of Life Sciences, University of KwaZulu-Natal, Durban, South Africa; 4 Department of Genetics, Evolution and Environment, University College London, London, UK; 5 Wildlife Conservation Research Unit, Department of Zoology, The Recanati‐Kaplan Centre, University of Oxford, Tubney, UK; 6 Department of Ecology, Evolution and Behavior, University of Minnesota, St. Paul, MN USA

**Keywords:** Biogeography, Conservation biology

## Abstract

African lions (*Panthera leo*) and African savanna (*Loxodonta africana*) and forest (*L. cyclotis*) elephants pose threats to people, crops, and livestock, and are themselves threatened with extinction. Here, we map these human-wildlife conflicts across Africa. Eighty-two percent of sites containing lions and elephants are adjacent to areas with considerable human pressure. Areas at severe risk of conflict (defined as high densities of humans, crops, and cattle) comprise 9% of the perimeter of these species’ ranges and are found in 18 countries hosting, respectively, ~ 74% and 41% of African lion and elephant populations. Although a variety of alternative conflict-mitigation strategies could be deployed, we focus on assessing the potential of high-quality mitigation fences. Our spatial and economic assessments suggest that investments in the construction and maintenance of strategically located mitigation fences would be a cost-effective strategy to support local communities, protect people from dangerous wildlife, and prevent further declines in lion and elephant populations.

## Abstract (web summary)

Growing human population density and farming expansion are fuelling human-wildlife conflict. Here the authors map spatial conflict with lions and elephants across Africa, identify high-risk areas, and estimate the cost-effectiveness of mitigation fences.

## Introduction

Current rates of species extinction are unprecedented^1^ and are destined to increase without adequate conservation actions^2^. Large-bodied mammals have suffered significant population declines over the past century and are further threatened by continued habitat loss, unsustainable use, and human–wildlife conflict^3^. The transformation of natural habitat to agriculture and intensive livestock husbandry has not only contracted these species’ ranges and largely restricted their distribution to the confines of protected areas^4–6^, but the closer proximity of human activity to wildlife has also increased the dangers posed to people, livestock, and crops^7^. Human–wildlife conflict involves the tangible and/or perceived impacts of wildlife on people^8^, including human injury and death^9^, direct and indirect economic damage to crops, livestock, and property^10^, food insecurity^11^, and diminished psychological wellbeing^12^. Unmitigated conflict decreases local support for biodiversity conservation^13^ and frequently escalates into retaliatory killing of wildlife^14,15^. Implementing effective human–wildlife conflict-mitigation strategies has, therefore, become a growing priority for engaging rural communities and preventing localized wildlife extinctions. Mitigation efforts can be broadly classified into tactics that directly or indirectly target wildlife (e.g., culling or translocating problem animals vs. noxious stimuli to deter crop-raiding elephants^16^, or improved livestock husbandry to reduce lion predation^17^), whereas other approaches attempt to increase tolerance to economic losses inflicted by wildlife (e.g., compensation schemes^18^, performance–payment schemes, and increased benefits to local communities from wildlife-based tourism; see ref. ^8^ for a review), although the large-scale effectiveness of most of these efforts remains equivocal^8,19,20^.

Africa is one of the last global strongholds for the conservation of large carnivores and herbivores^5,7^. However, Africa’s human population is projected to grow from the current 1.2 billion people to nearly two billion by the end of the century^21^ and Africa is also the centre of large-scale agricultural investments for the purposes of food and biofuel production^22^. Unsurprisingly, numerous parts of Africa have been identified as major hotspots of human pressure on biodiversity^23,24^ and pressures will likely intensify further as a result of future pandemics, political instability, or armed conflicts that hinder wildlife-based tourism, reduce effective conservation funding, and undermine national economies^25^. The continent-wide conservation challenges of human–wildlife conflict are encapsulated by the iconic African lion and the African savanna and forest elephants (hereafter referred to as elephants), which have all experienced extensive range contractions^4,5^ and suffered local extinctions and significant population declines throughout their ranges^26,27^, largely owing to (i) habitat loss^28^, (ii) unsustainable hunting^26,29^, (iii) retaliatory and preemptive killing to protect humans, livestock, and crops^8^, and (iv) extensive prey depletion (for lions)^30^.

Recent evidence suggests that African lion and elephant populations are persisting, or even increasing, in areas where conservation budgets are adequate and/or mitigation fences successfully prevent conflict with humans^19,27,31,32^. According to protected area managers, mitigation fences are essential along boundaries with the highest human, crop, and livestock densities, as alternative mitigation strategies are often ineffective^33^ (but see ref. ^20^ on the lack of quantitative comparisons about the utility of the alternatives) and mitigation fences are currently found in at least ten African countries, despite the costs of attaining the necessary standards^19,33^. However, a number of conservationists have expressed opposition to fencing on the grounds that large-scale barriers have often disrupted wildlife movements and decreased landscape connectivity in the past, and that these impacts will be likely to intensify as species respond to climate change^34^. However, these concerns were largely inspired by the widespread deployment of veterinary fences in southern Africa, where barriers were erected to prevent disease transmission from wildlife to livestock with little regard for their ecological impacts on migratory wildlife species^35^. Thus, the ongoing debate on the costs and benefits of fencing for both people and wildlife should turn its focus to identifying boundaries where fencing can be a financially sustainable strategy for preventing human–wildlife conflict, while minimizing any negative conservation impacts^36^.

Here we identify the areas that are most at risk for conflicts and estimate the associated return on investment of building and maintaining mitigation fences. Our analysis combines the most up-to-date information on the distribution of lions and elephants with spatial information on human population density, cropland, and cattle density, as these are considered to be the major drivers of human–wildlife conflict in Africa^4,8^. Conflict decreases with distance from protected areas^37,38^; thus, we identify areas on the perimeter of the ranges of lions and elephants that are within 10 km of the highest densities of humans, cattle, and crops. To avoid interrupting ecological processes such as migrations and/or causing unintended consequences to other biodiversity (e.g., habitat fragmentation), we extended the species ranges to include adjacent protected areas that currently lack lions and elephants, but were once part of their historical distribution (Supplementary Fig. 1). We identify a set of socio-economic and political variables that affect lion and elephant populations in each area (Supplementary Table 1), consider whether proposed fence lines would affect other migratory mammals, and estimate the associated equivalent annual annuity (EAA; i.e., the constant annual cash flow potentially generated by fencing over its lifespan with the net present value (NPV) being calculated on an annualized basis^39^), to determine the return on investment of building and subsequently maintaining the necessary mitigation fences at standards that can successfully restrict lions and elephants, and reduce cattle loss, crop damage, and human injury or death. It is noteworthy that our protocol identifies high human-occupancy areas that already block wildlife movements and otherwise disrupt large-scale ecosystem processes^40–43^, so the erection of mitigation fences would mostly act to separate humans from dangerous wildlife, but we nevertheless examine whether such barriers would inflict substantial further ecological impacts. Also note that the economic analyses presented here refer to high-standard fences built along the perimeter of conservation areas that effectively restrict lions and elephants. Given that the associated construction costs for fencing are the highest of any mitigation strategy currently in use or being field tested, our analysis embeds these expenses into an economic framework and asks where such expenditures would be cost-effective. Supplementary Fig. 2 provides a flowchart of the analysis; full details are provided in the ‘Methods’. We find that 82% of all sites containing lions and elephants are adjacent to areas with considerable human pressure. Areas at severe risk of conflict (adjacent to high densities of humans + crops + cattle) comprise 9% of the perimeter of these species’ ranges. These worst affected areas are found in a total of 18 countries that respectively host ~74% and 41% of African lion and elephant populations. Although a variety of conflict-mitigation strategies could be deployed to address this issue, we show how mitigation fences would provide considerable return on investment via reduced cattle loss and crop damage, especially in Tanzania, Ethiopia, and Kenya. Attention should be paid to prevent further habitat fragmentation for migratory species traversing the worst affected areas.

## Results

**Fig. 1:**
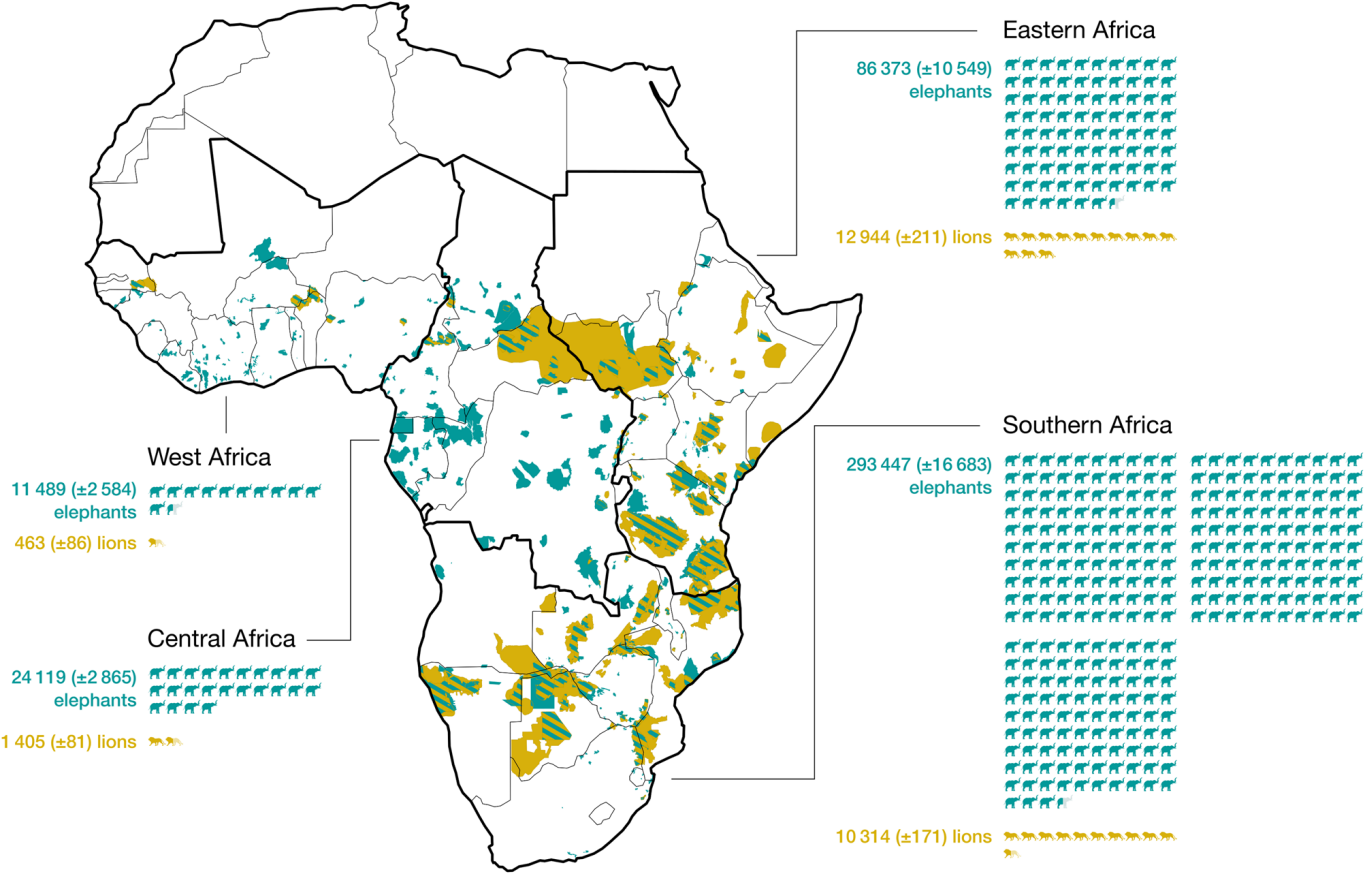
Distribution of African lions (*Panthera leo*) and African elephants (*Loxodonta Africana* and *Loxodonta cyclotis*), and regional contribution to their conservation. Lion ranges are in orange, whereas elephant ranges are in turquoise. Areas hatched in orange and turquoise represent overlapping species ranges. Each animal icon is equivalent to 1000 individuals. Values in parenthesis refer to 95% confidence intervals. Silhouette for lion is in the public domain and available from phylopic.org and silhouette for elephant is free for personal and commercial purpose from www.flaticon.com.

Based on survey estimates, there are ~25,125 (±549) lions and 415,428 (±20,112) elephants left in Africa (Fig. 1, Supplementary Fig. 3, and Supplementary Table 2). Human population density is the most important factor predicting population numbers of both lions and elephants (Supplementary Fig. 4 and Supplementary Table 3): these species are most abundant at localities where human population density is lowest. At a national scale, lion populations are higher in countries with higher conservation expenditures and elephant numbers are higher in countries with higher gross domestic product per capita (Supplementary Fig. 4 and Supplementary Table 3).

**Fig. 2:**
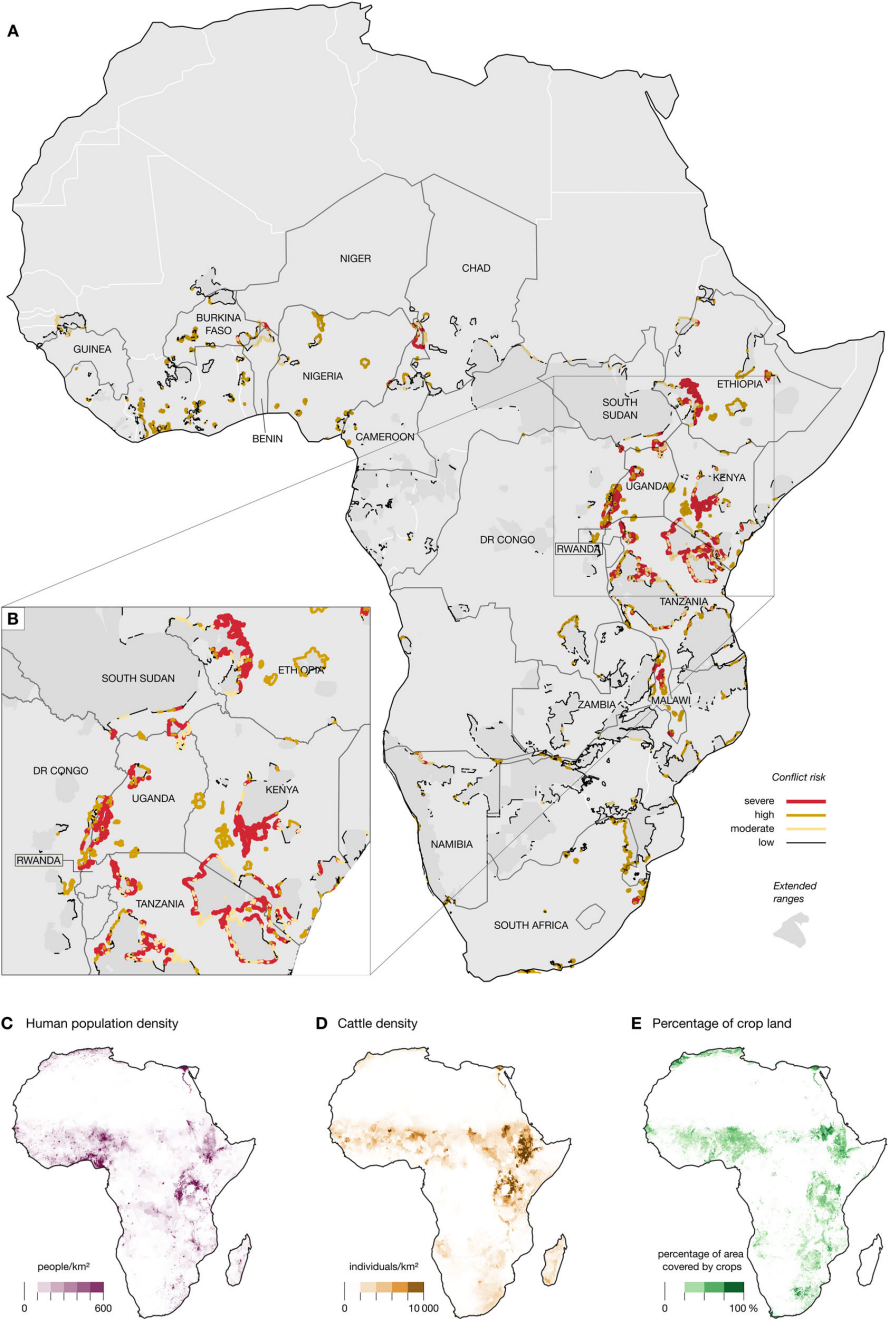
Areas at risk of conflict on the extended ranges of African lions (*Panthera leo*) and African elephants (*Loxodonta africana* and *Loxodonta cyclotis*). **A** Areas at risk of conflict across all of Africa. Extended range of elephants and lions are in dark grey. Definitions of severe, high, moderate, and low risk of conflict are given in the legend to Table 1. **B** Areas at risk of conflict in East Africa. **C** Human population density, **D** cattle density, and **E** proportion of cropland maps. See handling of uncertainty over spatial mapping in Supplementary Figs. 5 and 6.

Overall, 82% of all sites (i.e., protected and other conservation areas) containing lions and elephants in Africa are adjacent to areas with substantial human pressures (Fig. 2). About 60% of the perimeter of these ranges is adjacent to areas with high densities of human population, crops, or cattle (Table 1). Nine percent of the perimeter (totalling about 10,000–12,000 km) is at severe risk of conflict because of the co-occurrence of all three human pressures and these areas are distributed across 18 different countries (Fig. 2 and Table 1). These 18 countries are also among the most important for lion and elephant conservation, hosting ~74% and 41% of the entire lion and elephant populations, respectively. Another 10% of the perimeter, distributed across 26 countries, is at high risk of conflict, as they contain areas facing high human population density plus either high crop density for elephants or high cattle density for lions (Fig. 2). Countries with severe and high risks of conflict host 95% of Africa’s lions and 66% of Africa’s elephants.

**Table 1:** Percentage of the extended ranges of African lions (*Panthera leo*) and African elephants (*Loxodonta africana* and *Loxodonta cyclotis*) under different risk of conflict with humans.

Country	Extended-range perimeter (km)	% at low risk	% at moderate risk	% at high risk	% severe risk	% at risk
Rwanda	299	0	1	0	99	100
Uganda	3036	3	9	8	50	70
Kenya	7293	10	8	4	36	57
Tanzania	10,697	32	20	12	29	93
Ethiopia	8116	10	6	2	26	44
Malawi	2090	5	1	65	23	94
Niger	315	3	37	0	21	60
South Sudan	2662	20	5	3	11	39
Cameroon	5086	19	5	6	6	36
Benin	649	31	64	1	4	100
South Africa	5326	31	0	52	3	85
Democratic Republic of Congo	11,846	12	0	11	2	26
Chad	3976	62	9	0	1	72
Burkina Faso	2399	16	2	18	1	37
Guinea	830	30	13	3	1	47
Nigeria	2882	13	5	30	1	49
Namibia	6455	38	3	8	1	49
Zambia	7988	72	2	3	1	78
Angola	2805	44	0	5	0	49
Botswana	3391	36	1	0	0	37
Central African Republic	1792	14	0	0	0	14
Congo	5393	16	0	0	0	16
Côte d’Ivoire	5105	30	0	26	0	55
Equatorial Guinea	181	71	0	0	0	71
Eritrea	451	2	0	0	0	2
Eswatini	287	0	0	1	0	1
Gabon	4732	27	0	0	0	27
Ghana	2802	30	0	10	0	40
Guinea-Bissau	373	43	0	0	0	43
Liberia	1736	4	0	0	0	4
Mali	1378	68	1	0	0	69
Mozambique	7697	70	1	10	0	81
Senegal	763	58	33	0	0	91
Sierra Leone	556	18	0	4	0	23
Somalia	1060	16	2	0	0	18
Sudan	884	5	37	0	0	43
Togo	718	41	0	36	0	77
Zimbabwe	6624	87	3	4	0	94

Note: Classifications are based on the extended range of each species. Severe risk is where elephant and lion extended ranges overlap with the highest human population, crop, and cattle densities; high risk includes the highest human population and crop densities for elephants and the highest human population and cattle densities for lions; moderate risk involves the highest crop density for elephants and highest cattle density for lions; low risk includes only one human pressure, i.e., highest human population density, highest crop density, or highest cattle density. See estimates of variability in total length of perimeter and handling of uncertainty over spatial mapping in Supplementary Tables 4 and 5, and in Supplementary Figs. 5 and 6.

Sensitivity analyses confirmed the same countries with areas at severe risk regardless of the buffer distances used in the spatial analyses (Supplementary Fig. 5 and Supplementary Table 4), and the locations of severe- and high-risk areas of conflict are robust to randomly varying the distances between the species-range perimeter and the human pressure maps (Supplementary Fig. 6 and Supplementary Table 5). It is noteworthy that the presence of lions and elephants is more certain in the areas identified as being at severe risk of conflict (Supplementary Fig. 7). In addition, mitigation fences in the severe conflict areas would not increase habitat fragmentation for most of the associated migratory mammals (with the exception of a slight increase of fragmentation for Grévy’s zebra *Equus grevyi* and Thomson’s gazelle *Eudorcas thomsonii*) (see Supplementary Table 6). Furthermore, it is worth noting that most countries with severe and high risk of conflict are also likely to experience the highest human population growth by 2100 (Supplementary Table 7).

**Fig. 3:**
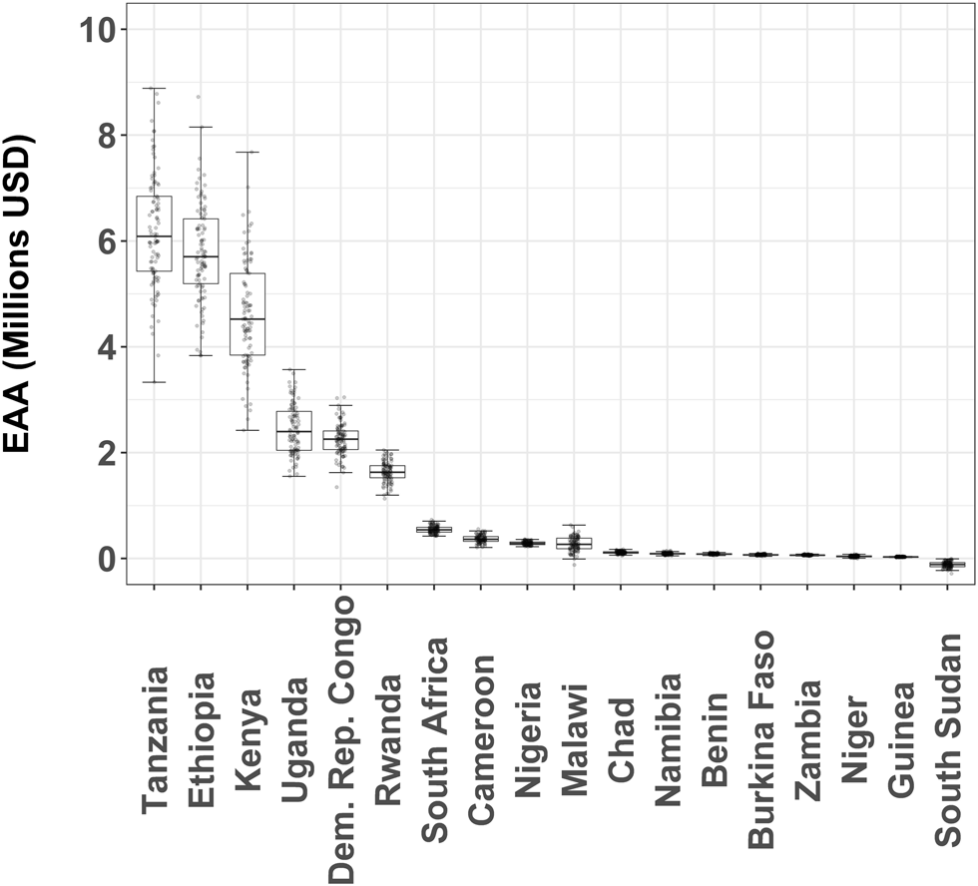
Boxplot of the equivalent annual annuity (EAA) of building and maintaining mitigation fences in areas at severe risk of conflict between humans and African lions (*Panthera leo*) and African elephants (*Loxodonta africana* and *Loxodonta cyclotis*). Whiskers represent range from minimum to maximum, box indicates 25 and 75 percentile, and horizontal line represents median. Plotted dots represent 100 EAA values calculated by varying all economic model parameters randomly across ±10% of the values of each parameter. Dots outside the whisker boundaries are outliers. Calculations do not consider the additional benefit of reducing costs of human injury or death.

Although the construction and maintenance costs of mitigation fences at such a large scale might seem prohibitively expensive, elephants and lions inflict considerable damages to crops and livestock in many parts of Africa^8,19,44,45^. Installing and maintaining mitigation fences would likely provide a net return on investment in all 18 countries with areas at severe risk of conflict with the exception of South Sudan (Fig. 3), with Tanzania, Ethiopia, and Kenya being the countries where investments in mitigation fences around such areas would be most cost-effective in terms of reducing cattle loss and crop damage (Fig. 3 and Supplementary Fig. 8). In contrast, installing and maintaining mitigation fences in high conflict-risk areas would seldom generate sufficient return on investment and, therefore, other mitigation strategies would be preferred (Supplementary Fig. 9). When considering per capita benefits, installing and maintaining mitigation fences around severe conflict areas could potentially provide the highest return on investment for local people living in Benin, South Africa, and Zambia (Supplementary Table 8).

## Discussion

Our results show that lions are at greater risk of conflict with humans than are elephants. Without adequately funded conservation actions, there are likely to be serious future risks of population declines or local extinctions that will affect 74% of the entire lion population. Elephants, on the other hand, still occur in relatively high numbers in low human-occupancy areas^46^. However, Africa’s projected human population growth will almost certainly spread the severe conflict risks to include areas currently classified as only high risk, and population declines or local extinctions of lions and elephants will ultimately affect national economies in countries that depend heavily upon revenue generated from wildlife-based tourism and sustainable utilization^47,48^.

Our results also highlight that, in countries with areas at severe risk of conflict, mitigation fences are an economically sustainable strategy that can potentially be used to help reduce human–wildlife conflict at large scales. Building such fences in severe-risk conflict areas could provide an economically viable action to reduce crop damage and livestock losses; reductions in crop damage and cattle loss could, in turn, enhance tolerance for lions and elephants^8^. By contrast, our results suggest that the return on investment from expensive fencing strategies might not repay themselves in countries with lower levels of human–wildlife conflict. In these cases, alternative strategies, e.g., those that rely on human-dimension approaches to enhance co-existence^8^ between humans and wildlife, would potentially be a more cost-efficient solution to mitigating conflict, assuming they can be both effective and sustainable in perpetuity. It is noteworthy, though, that our analysis only considers the direct economic benefits of fencing but does not take into consideration benefits from preventing human deaths or injuries, or mitigating less tangible psychological effects, fears and anxieties that cannot easily be monetized^49^, all of which would be reduced by fencing even in moderately affected areas. On the other hand, our analysis neglects the economic costs imposed on local people by mitigation fences (e.g., restrictions on access to protected areas), although these could be minimized through permit systems and strategically placed access points.

Although large-scale agriculture and high-density human settlements often disrupt animal movements as effectively as mitigation fences^41–43^, attention should clearly be paid to risks of more completely interrupting ecological processes such as mammalian migrations (see ref. ^50^). Our results highlight the potential for Grévy’s zebra and Thomson’s gazelle to be affected by building mitigation fences in the severe-risk areas without safeguards to prevent blockage of migration corridors. Indeed, fine-scale studies of animal movements from collared animals should ideally be employed to prevent placing fences in areas that would obstruct such migrations^51^. Future studies should also assess how mitigation fences would affect other taxonomic groups, such as invertebrates and plants, and ecological processes (e.g., seed dispersal)^34,52,53^, and investigate the measures that could reduce any local impacts.

Interestingly, several areas of lion and elephant habitat in South Africa are under relatively low risk of conflict with humans compared to other parts of Africa. Thus, the country that first utilized fencing for conservation has the potential to re-open some of its wildlife areas to restore large-scale ecosystem processes, continuing a pattern started in 1993 when fences along the western boundary of Kruger National Park were dropped to annex 1800 km^2^ of wildlife habitat in the associated private nature reserves. Furthermore, extensive mammalian migrations could potentially be restored by removing veterinary fences in Botswana and Namibia, which were erected in the 1970s to reduce disease transmission from wildlife to livestock (e.g., in the Kavango Zambezi Transfrontier Conservation Area). We emphasize that any decision to erect a mitigation fence should be premised on reducing human–wildlife conflict rather than arbitrarily restraining natural movements of animals across extensive landscapes; existing fences should also be interrogated for their purpose and function, as well as their unintended consequences on biodiversity conservation and sustainable development.

As with any large-scale spatial analysis, data quality should be taken into consideration. First, our species-range maps represent coarse-resolution distributional boundaries rather than fine-resolution edges of suitable habitat. However, we were partly able to address this issue by using population sizes of lions and elephants within each area. Second, the financial costs of conflict mitigation are likely to vary geographically based on physical and socio-economic factors. We used the most up-to-date fencing costs wherever possible throughout sub-Saharan Africa^33^, but this information is not available in countries where mitigation fences do not yet exist. Our calculation of the EAA of fencing utilized a variety of country-specific information of market prices, crop yields, etc., but our approach assumes that the financial benefits will be returned to local stakeholders and not to the donors/agencies who would invest in fence construction in the first place. Third, our results should only be viewed as a continent-wide assessment rather than as a precise blueprint for implementing local-scale mitigations. The latter would require on-the-ground validation and adaptation to local circumstances, especially where species ranges extend beyond protected area boundaries and into community land; local-level consultations would be essential for promoting acceptance and support for these strategies rather than risking additional disputes between wildlife managers and local communities (Supplementary Fig. 7).

We consider this study as foundational for informing future work that could holistically integrate human dimensions of human–wildlife conflict by inspiring collaborations with local communities to explore their willingness to accept or reject hard strategies such as mitigation fences^8,54^. The intention of a mitigation strategy such as fencing should not be to completely exclude people from access to parks but should be negotiated by collective agreements. For example, access gates could facilitate access of local communities to water and other natural resources, as well as for various cultural purposes^55^. Areas with effective land-sharing and pre-existing community benefits from wildlife^56,57^ could use potential fence lines as metaphorical tools for discussion and negotiations among stakeholders. A central goal of conflict mitigation is to prevent lion and elephant attacks^37^; reducing these threats would not only enhance human wellbeing in terms of lives saved but also improve mental health (sensu^12^). For example, conflict with elephants in Botswana raised concerns in local people as to food security, safety, and mobility^58^. Additional costs, such as time expenditures on crop protection or livestock guarding, and risks of infectious diseases^12^ could also be reduced.

In conclusion, we stress the importance of immediate action to minimize current and future human–wildlife conflict in Africa. Areas of intensive human pressure already produce hard boundaries around remaining areas of natural habitat, thus reliance on strategies such as mitigation fencing would merely reflect the reality of conserving large, dangerous wildlife species in human-dominated landscapes. Effective conflict mitigation could potentially motivate improved conservation of elephants and lions, while retaining the socio-economic benefits that flow from intact wildlife systems that still host substantial numbers of lions and elephants. The need for substantial investments has never been more urgent, as the coronavirus disease 2019 crisis has drastically reduced the benefits of wildlife-based tourism in some regions^25,59^, likely increasing costs of living with lions and elephants, and exacerbating conflict with humans. Our pan-African spatial assessment of human–wildlife conflict provides an important starting point for informing future research and conservation planning at finer geographical scales.

## Methods

After preprocessing the data, methods consisted of spatial analyses to map areas at risk of conflict; statistical analyses to identify the most important factors affecting lion and elephant population numbers; economic analyses to estimate the EAA of building and maintaining mitigation fences in areas under severe and high risk of conflict, and fragmentation analyses to assess the impact of fences on migratory mammal species. We describe each step in detail below (see Supplementary Fig. 2 for a flowchart of the analysis). All spatial data were converted to vectors for analysis to reduce commission errors (when a species is mistakenly thought to be present) when converting the species-range maps from vector to raster. Data preprocessing was carried out using the open source database PostgreSQL 11.4 (https://www.postgresql.org/about/) with the GIS extensions of PostGIS 2.5 (https://postgis.net/); conflict mapping and range fragmentation analyses used PostgreSQL 11.4 and PostGIS 2.5, and Python v. 3.7.0^60^; statistical and economic analyses used R v. 3.6.0^61^; sensitivity analyses used PostgreSQL 11.4 and PostGIS 2.5, and Python v. 3.7.0^60^ and R v. 3.6.0^61^.

### Preprocessing

#### Human pressures

Human pressure layers were independently generated from this study. We used Gridded Population of the World Version 4 (GPWv4) as a layer for human population density^62^. GPWv4 is a minimally modelled data set consisting of estimates of human population (number of persons per raster grid cell) based on non-spatial population data (i.e., tabular counts of population listed by administrative area) and spatially explicit administrative boundary data. Population input data are collected at the most detailed spatial resolution available from the results of the 2010 round of Population and Housing Censuses. The input data are then extrapolated to 2020 using calculated growth rates to produce future population estimates. A proportional allocation gridding algorithm, utilizing ~13.5 million national and subnational administrative units, assigned population counts to 30 arcsecond (~1 km at the equator) grid cells. The population density rasters were created by dividing the population count raster for a given target year by the land-area raster.

We used the most recent version of the Gridded Livestock of the World database^63^, reflecting the compiled and harmonized subnational livestock distribution data for 2010, to extract information on cattle density. The data set provides global population densities of cattle, buffaloes, horses, sheep, goats, pigs, chickens, and ducks in each land pixel at a spatial resolution of 0.083333 decimal degrees (~10 km at the equator). Detailed livestock census statistics are mined from agricultural yearbooks or through direct contacts with ministries or statistical bureaus. The census statistics are usually found in the form of numbers per administrative unit that must be linked to corresponding geographic information system boundaries. Densities are estimated in each census polygon by dividing the number of animals from the census by the surface area of the administrative unit polygon (estimated in an Albert equal-area projection), corrected by a mask excluding unsuitable areas. Livestock densities were then extracted from the subnational census data and were used as the dependent variable in Random Forest models to estimate a density value in each pixel, based on raster predictor variables.

We used spatially detailed crop maps available from the Copernicus Global Land Cover map at ~0.001° (~100 m) resolution^64^. The land-cover map is a discrete map with ten continuous cover fractions (nine base land-cover classes and seasonal water) to provide spatial information about land for a diversity of applications, including biodiversity conservation. Cropland (as percentage of 100 m pixel that is covered by cropland) refers to cultivated and managed agriculture, but does not include perennial woody crops that are classified under the appropriate forest or shrub land-cover type^64^. Cropland also refers to both irrigated and rainfed agriculture. The land-cover map was generated by compiling the 5-daily PROBA-V multi-spectral image data with a Ground Sampling Distance of ~0.001° as the primary earth observation data and PROBA-V UTM daily multi-spectral image data with a Ground Sampling Distance of ~0.003° (~300 m) as the secondary earth observation data. Next, the 5-daily PROBA-V 100 m and daily 300 m datasets were fused using a Kalman filtering approach. The global overall accuracy of the product for the base year 2015 was calculated through an independent pre-validation and reached 80%.

#### Species-range maps

Updated range maps showing current distribution for lions and elephants were provided by the International Union for Conservation of Nature (IUCN) Cat and African Elephant Specialist Groups^65^. In addition to the range maps, the specialist groups provided information on the number of African lions (2018) and elephants (2016) within sites where they are still extant. We also obtained species-range maps for all terrestrial mammal species in orders Cetartiodactyla, Perissodactyla, Primates, and Carnivora occurring in Africa from the IUCN Red List portal (www.iucnredlist.org/). Mammal species in these orders include migratory mammal species (e.g., the common wildebeest *Connochaetes taurinus*), which might be negatively affected by mitigation fences, e.g., by potentially blocking migratory routes.

#### Protected areas

The data on protected areas were based on the May 2019 release of the World Database on Protected Areas^66^ (retrieved from http://www.protectedplanet.net). To prevent overestimation of the area coverage of protected areas caused by overlapping designations, we merged polygons into a single layer. We only included in the analysis IUCN categories Ia (Strict Nature Reserve), Ib (Wilderness Area), II (National Park), III (Natural Monument or Feature), and IV (Habitat/Species Management Area), because we wanted to prevent fences from excluding people from protected areas that had been modified by the interaction of nature and people over time (e.g., V, Protected Landscape/Seascape).

### Mapping potential risk of conflict

A database on the spatial distribution of conflict locations between humans and lions and elephants is not available across Africa. We therefore mapped the most prominent factors known to affect conflict: human population density (for both lion and elephant), crop raiding (elephants), and cattle killing (lions)^8^. Furthermore, spatial modelling of range contractions in carnivores showed that contractions were significantly more likely in regions with high rural human population density, cattle density, and/or cropland^4^. Therefore, we only retained areas where human, cattle, and crop densities were in the first decile (in our case, the first decile is the decile with the highest human population, crop, and cattle densities) by PostgreSQL/PostGIS. Using only the highest decile likely resulted in a conservative map of spatial conflict.

We further classified areas at the highest potential conflict into low, moderate, high, or severe risk of conflict. Specifically, areas at severe risk of conflict are those where the highest human population, crop, and cattle densities all overlap; areas at high risk of conflict are those with overlaps between the highest densities of human population and either crops or cattle; areas at moderate risk of conflict are the areas where the highest crop and cattle densities overlap; and areas at low risk of conflict are those with only one human pressure, i.e., the highest human population, or crop, or cattle density. The remainder was considered as being at no risk of conflict, as it did not meet any of the above criteria, but note the conservative nature of our analysis (see above).

The lion and elephant range maps and the protected area layer were intersected to select all protected areas that contain parts of lion and elephant range and/or were adjacent to the species-range maps. The identified protected areas were then merged with the species-range maps to create a new extended range layer (see for an example in Supplementary Fig. 1). These extended range maps were used (i) to identify potential areas where lions and elephants could be restored, and (ii) to avoid interrupting ecological processes (e.g., migrations) and/or causing unintended consequences (e.g., fragment populations) to other biodiversity in neighbouring protected areas.

We then identified areas at risk of conflict by intersecting the extended range map layer for lions and elephants with the classified conflict map. In all cases, the intersections were carried out so that the classified conflict areas were either adjacent to, or within a distance of 10 km from, the edge of the extended range map layer. We set this distance to consider the wide-ranging behaviour of both lions and elephants, to account for the fact that conflict decreases at greater distances from protected area boundaries^37,38^, and to account for the fact that future human pressures will likely increase before conservation actions take place^2^.

We assessed how robust our results were to commission (where human pressure is mistakenly assumed to exist) and omission (where human pressure is mistakenly assumed to be absent) errors in the human pressure maps by carrying out a sensitivity analysis that randomly varied the distances between the extended range maps and the human pressure maps. We first used Latin hypercube sampling, which is a form of sampling used to reduce the number of runs necessary for a Monte Carlo simulation to achieve a reasonably accurate random distribution^67^, to randomly vary 100 times the distance values between the extended range and human pressure maps. Specifically, we divided the low, moderate, high, and severe conflict lines into 100 m segments, calculated the minimum distance for each segment to human pressure within a 10, 20, and 30 km buffer distance from the edge of the extended range map layer, and then randomly varied that distance 100 times across ±10% of the value. We then averaged the resulting 100 randomly created distance values for each segment and identified which segments fell outside of the analyzed buffer distances of 10, 20, and 30 km. We tested for 20 and 30 km buffer distances, as we wanted to assess the variability of the fencing distance to different buffer sizes. We also estimated the certainty of lion and elephant presence by identifying segments of the perimeter of the range maps of lion and elephant that overlapped with protected areas. We did this as we had information on certain presence of both species from within protected areas, as opposed to areas extending outside of protected areas.

### Statistical analyses

We used an information theoretic approach^68^ and Bayesian information criterion to calculate statistical models. We used generalized linear mixed models with a negative‐binomial error distribution to account for over-dispersed count data and a log‐link function to examine factors affecting lion (*n* = 77) and elephant (*n* = 191) population sizes in Africa. Generalized linear mixed models were fitted with both random and fixed effects, to capture the data structure. Country was included as a random intercept to represent the hierarchical structure of the data. All variables listed in Supplementary Table 8 were fitted as fixed effects, i.e., with constant regression coefficients across countries. The site-specific variables were calculated only for sites where lions and elephants are currently present and not for the extended ranges. For transboundary sites that stretch across countries, we used the value for Gross Domestic Product, Conservation expenditure, and the Ibrahim Index of African Governance, for the country making the largest area contribution to the site. We compared and ranked models using the Bayesian information criterion^68^. To avoid multicollinearity among variables, we only selected variables with the strongest effect on population numbers that correlated at *r* < 0.7. Therefore, only one member of each pair that had a correlation >0.7 was selected as an input into the modelling process. We assessed each model’s relative probability, using Bayesian information criterion weights and the structural goodness-of-fit from the percentage of deviance explained by the model. We determined the magnitude and direction of the coefficients for the independent variables with multi-model averaging implemented in the R package *glmulti*^69^. The relative importance of each predictor variable was measured as the sum of the weights over the six top‐ranked models with Bayesian information criterion values closer to that of the best model containing the parameter of interest. Finally, we used a 10-fold cross-validation (a bootstrap resampling procedure using 1000 iterations) to assess the predictive ability of the top-ranked model.

### Range fragmentation analyses

We assessed how the proposed mitigation fences affected species-range connectivity by calculating the perimeter length-to-area ratio for mammal species in orders Cetartiodactyla, Perissodactyla, Primates, and Carnivora, whose ranges were identified as intersecting with areas at severe risk of conflict. Minimizing the perimeter length-to-area ratio is an important method of optimizing protected area design, resulting in compact reserves with high connectivity that can enhance persistence of the species. The smaller the ratio, the greater the clumping and connectivity of the species ranges. Specifically, we calculated the ratios of perimeter length to area for the ranges of 20 migratory mammalian species (i) under current conditions without fences and (ii) under future conditions where the identified mitigation fences would pass through their ranges. In the latter case, we used a 20 m buffer around the identified fences to account for further habitat clearance due to maintaining clearances around the fences for management purposes.

### Economic analyses

We used EAA to estimate the return on investment of building and maintaining mitigation fences to reduce cattle loss and crop damage. EAA calculates the constant annual cash flow generated by a project over its lifespan if it were an annuity and the annuity can then be compared to other projects of similar or different lifespan. Therefore, the measure potentially provides an important means for funders/donors to compare different investment opportunities. EAA is calculated by dividing the NPV of a project by the present value of annuity factor^39^. We started by calculating NPV in countries with areas at severe and high risk of conflict as:

1 $${{NPV}}={\sum }_{i}^{n}\frac{{R}_{i}}{{\left(1+d\right)}^{i}}-Z$$

where ${R}_{i}$ is net cash flow, $d$ is the discount rate specific to each country (Supplementary Table 9), *n* is the number of time periods, $i$ is the cash flow period, and $Z$ is the initial investment of building the fences. NPV was calculated over a 10-year investment period. ${R}_{i}$ was calculated as:

2 $${R}_{i}=B-C$$

where $B$ is the economic benefit derived from mitigation fences and $C$ is the cost of maintaining mitigation fences. The economic benefits of mitigation fences for countries with severe risk of conflict refer to the potential reduction in cattle loss (for lions) and crop damage (for elephants) derived from building fences:

3 $$B=L+E$$

where $L$ represents the economic benefits of reducing cattle loss and $E$ measures the economic benefits of reducing crop damage. For countries with high risk of conflict, the benefit ($B$) is derived from one or the other, i.e., $B$ = $L$ or $B$ = $E$.

4 $$L=v * w * P$$

where $v$ is the number of cattle that are not lost because of the presence of fences, $w$ is the average weight in kg of adult cattle in that country, and $P$ is the price of meat per kg paid to producers in that country in 2017 (data can be downloaded from http://www.fao.org/faostat/en/#data/PP). $v$ was calculated as the percentage of total cattle present in the 10 km buffer adjacent to severe and high conflict areas, which could potentially be killed, based on published estimates across Africa^45^. Estimates range from 0.8 to 2.6% of cattle losses, and we decided to use a conservative 1% loss in the analysis (see below for how we accounted for uncertainty in model parameters). $w$ *was based on the average weight* of an adult cow with estimates available at a regional level (west Africa: 262 kg; central Africa: 281 kg; east Africa: 283 kg; and southern Africa: 339 kg)^70^.

5 $$E=\left(d * A\right) * y * P$$

where $d$ is the percentage of crop area damaged by elephants; data are taken from published estimates (ranging from 0.2 to 4% and we used a conservative 1% in the analysis)^44^; $A$ is the total area in km^2^ available as crops in the 10 km buffer adjacent to the areas at severe and high risk of conflict; $y$ is the yield (ton/km^2^) for the crop known to be targeted by elephants (cassava, maize, millet, banana, sorghum, groundnuts)^44^, which covered the largest area size in that country in 2017 (data calculated from: http://www.fao.org/faostat/en/#data/PP); and $P$ is the price per ton paid to producers for that crop in that country in 2017 (data can be downloaded from http://www.fao.org/faostat/en/#data/PP). Although there might be several crops available within the buffer, this information is currently not available at the continental scale. Therefore, we decided to use the most common cultivated crop known to be targeted by elephants in each country.

The cost of maintaining mitigation fences ($C$) was calculated as:

6 $$C=f * c$$

where *f* is the fence length in that country and $c$ is the cost for maintaining the fence. We obtained cost estimates of building (*Z*) and maintaining $(c)$ the fences from Pekor et al.^33^. We used the median estimated current cost of USD 9522 per km for building fences and the median stated annual budget cost of USD 487 per km for adequate fence inspection and maintenance. This is the most up-to-date information validated through peer review on the costs (converted to 2017 USD) across Africa^33^. Cost estimates varied across surveyed conservation areas because of fence height and materials but included relevant costs of electrification and predator-proof structures^33^. The data were collected from 29 partially fenced (<90% of perimeter fenced) and 34 fully fenced (≥90% of perimeter fenced) protected areas, including, e.g., Kruger National Park in South Africa, across sub-Saharan Africa^33^.

Finally, we calculated EAA for each country as:

7 $${{EAA}}=\frac{{{NPV}}}{\frac{1-{\left(1+d\right)}^{-i}}{d}}$$

We used Latin hypercube sampling to vary all model parameters mentioned above randomly from within 100 partitions across ±10% of the values of each parameter and assess the uncertainty associated with model estimates on EAA. The partitioning across ±10% of the values of each parameter was deemed suitable to account for uncertainty over model parameters that were lacking estimates of variance. The resulting 100 EAA values for each country are shown in Fig. 3 and Supplementary Figs. 8 and 9.

### Reporting summary

Further information on research design is available in the Nature Research Reporting Summary linked to this article.

## Supplementary information

Supplementary Information

Reporting Summary

## Data Availability

Information on the distribution and population sizes of lion and elephant are available from the IUCN Cat and African Elephant Specialist Groups. The study used openly available datasets of Gridded Population of the World Version 4, Gridded Livestock of the World database, and crop maps available from the Copernicus Global Land Cover map with references provided in the ‘Methods’ section. Range maps for all terrestrial mammal species used in the fragmentation analyses are available from the IUCN Red List portal (www.iucnredlist.org/). The data on protected areas were available from the World Database on Protected Areas (http://www.protectedplanet.net). Data for the economic analyses are openly available from sources such as FAO and links are provided in the ‘Methods’ section. Our conflict-risk maps are available to download from https://etsin.fairdata.fi/dataset/d0ac647a-4d73-4117-89de-9d194215f948.

## Code availability

Code used for creating the conflict-risk maps is available at https://gitlab.com/helics-lab/spatial-analysis-human-wildlife-conflict.
