# Supplementary material for: A pan-African spatial assessment of human conflicts with lions and elephants
Source: Nat Commun. 2021 May 20;12:2978. doi: 10.1038/s41467-021-23283-w (PMC8138028; doi:10.1038/s41467-021-23283-w)
Supplement: Supplementary file 1 — Supplementary Information [file 41467_2021_23283_MOESM1_ESM.pdf]

A pan-African spatial assessment of human conflicts with lions and elephants

Enrico Di Minin [1,2,3*], Rob Slotow [3,4], Christoph Fink [1,2], Hans Bauer [5], and Craig Packer [3,6]

[1]Helsinki Lab of Interdisciplinary Conservation Science, Department of Geosciences and Geography, University of Helsinki, FI-00014, Finland; [2]Helsinki Institute of Sustainability Science (HELSUS), University of Helsinki, FI-00014, Finland; [3]School of Life Sciences, University of KwaZulu-Natal, Durban 4041, South Africa; [4]Department of Genetics, Evolution and Environment, University College, London, WC1E 6BT, UK; [5] Wildlife Conservation Research Unit, Department of Zoology, The Recanati-Kaplan Centre, University of Oxford, Tubney, UK; [6]Department of Ecology, Evolution and Behavior, University of Minnesota, St. Paul, MN, USA.

Supplementary Figures

Supplementary Figure 1. Example of extended range used in the analysis. African lion (*Panthera leo*) and African elephant (*Loxodonta africana* and *Loxodonta cyclotis*) ranges are in orange and turquoise, respectively. Protected areas are in green hatched. All overlapping or directly adjacent species range polygons and protected area polygons are combined into one continuous extended range polygon.

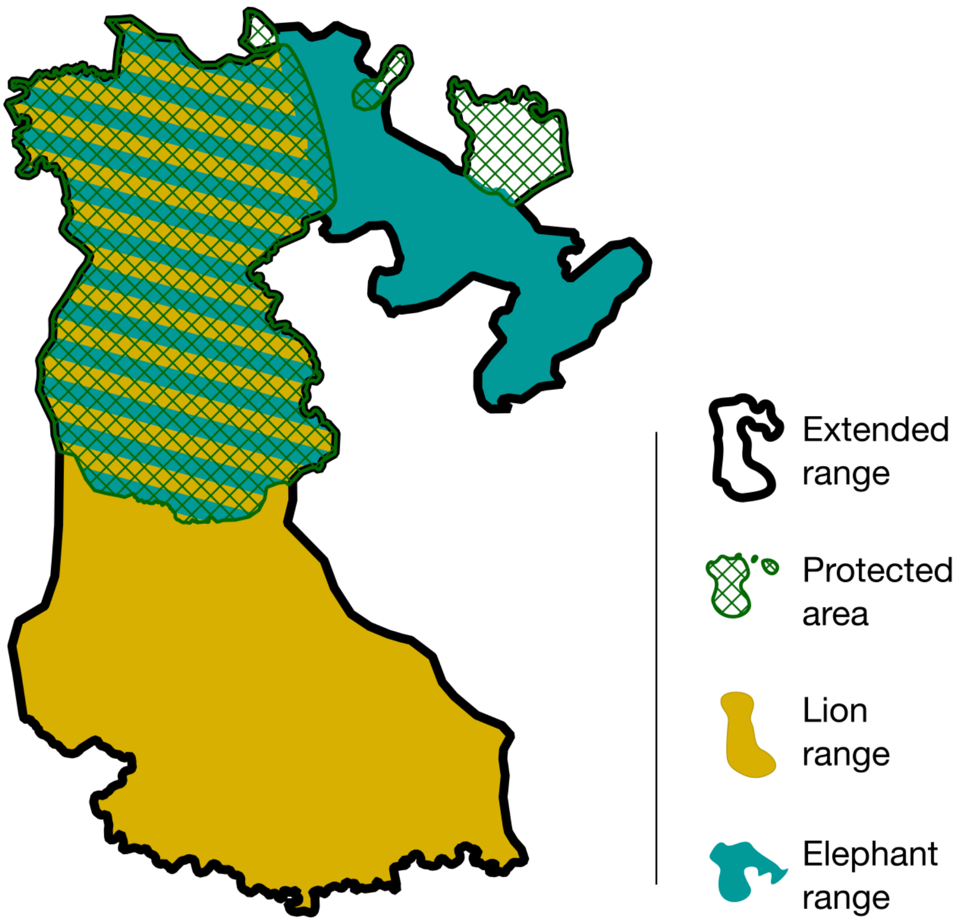

Supplementary Figure 2. Flowchart of the analysis (see Methods).

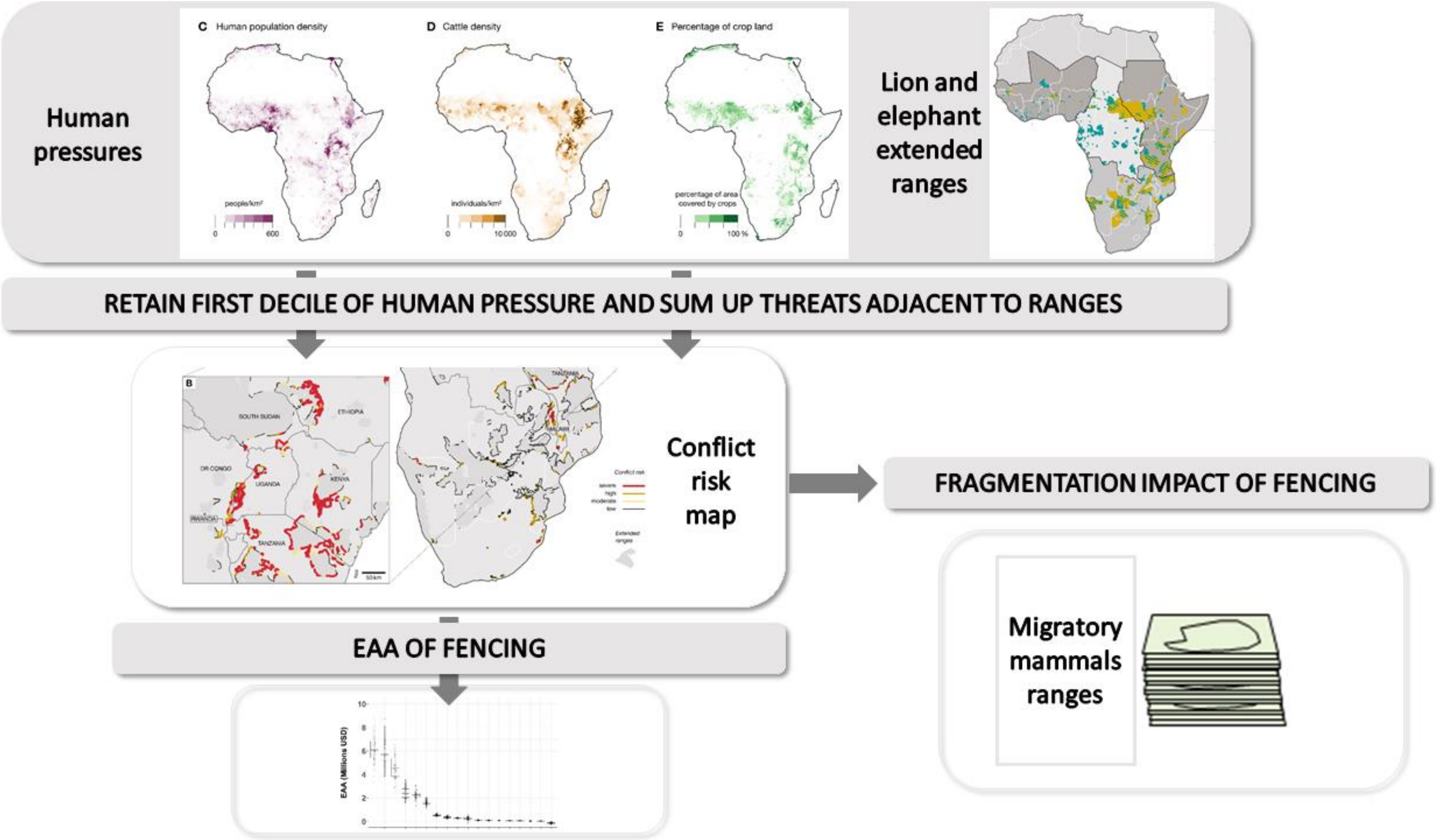

Supplementary Figure 3. Population densities of (A) African lions (*Panthera leo*) and (B) African elephants (*Loxodonta africana and Loxodonta cyclotis*).

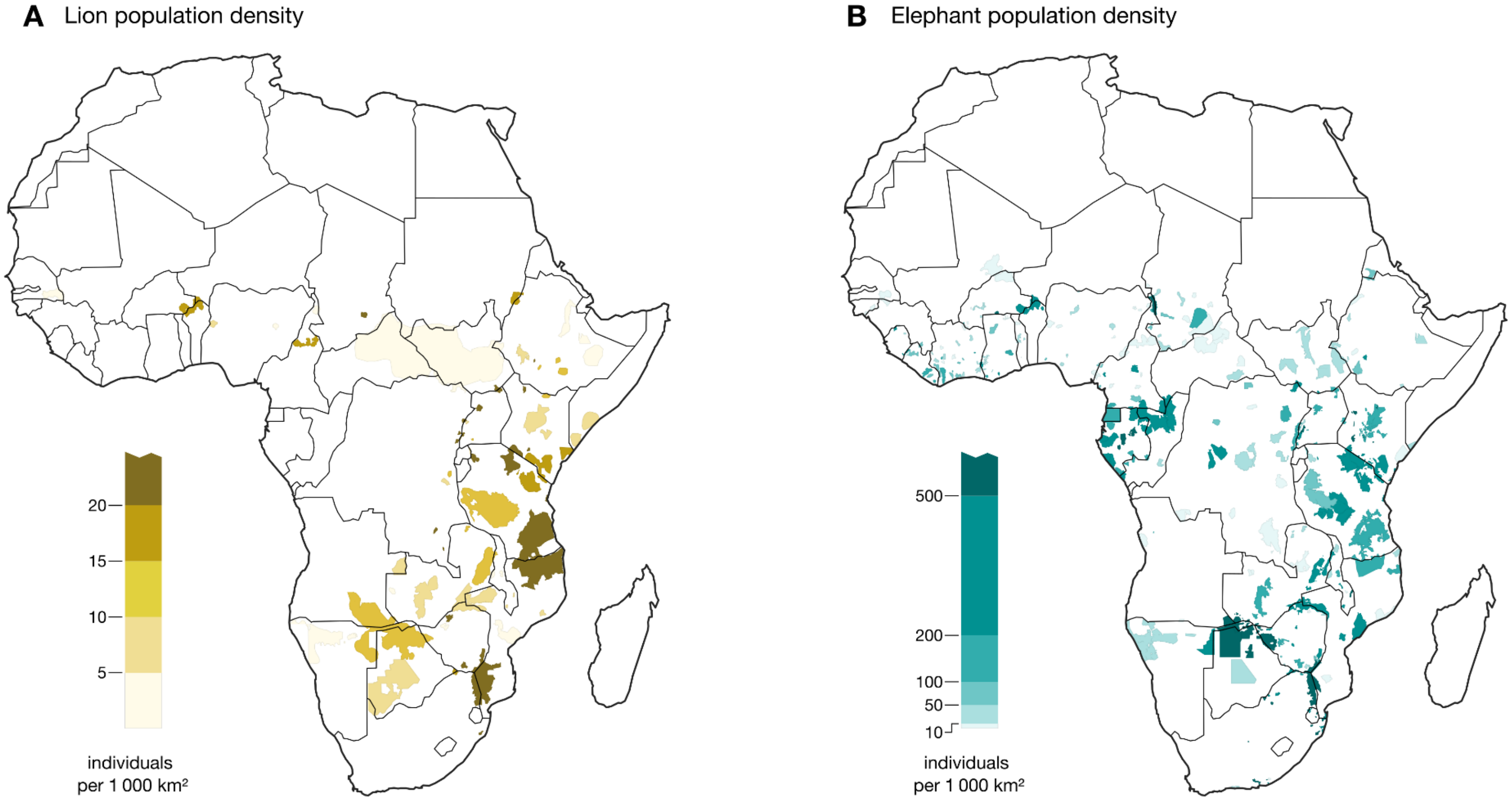

Supplementary Figure 4. Error Bar plot of regression coefficients (± standard error), showing the relative effects of five variables on (A) African lion (n = 77) and (B) elephant (n = 191) population numbers in Africa. hum_pop_dens = human population density; funding_cons = conservation expenditure; cattle_density = cattle density; governance = Ibrahim Index of African Governance; GDP = gross domestic product per capita; crop = proportion of crops. Stars show significant variables. For elephants, both significant variables had relative importance of 1.0; for lions, human population density had relative importance of 1.0, and conservation funding had relative importance of 0.85.

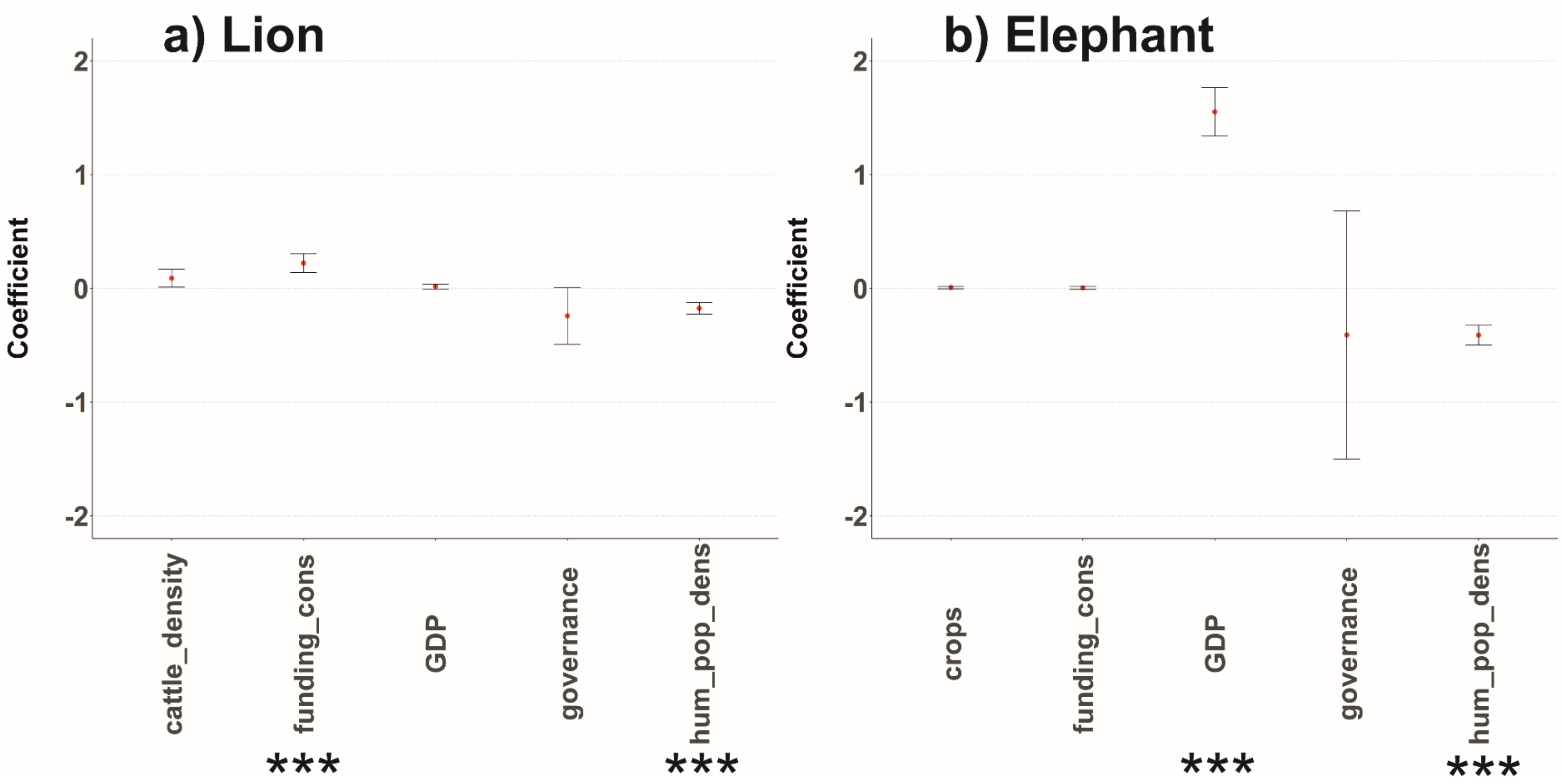

Supplementary Figure 5. Areas at risk of conflict on the extended ranges of African lions (*Panthera leo*) and African elephants (*Loxodonta africana* and *Loxodonta cyclotis*) at (A) 10, (B) 20, and (C) 30 km buffer distances.

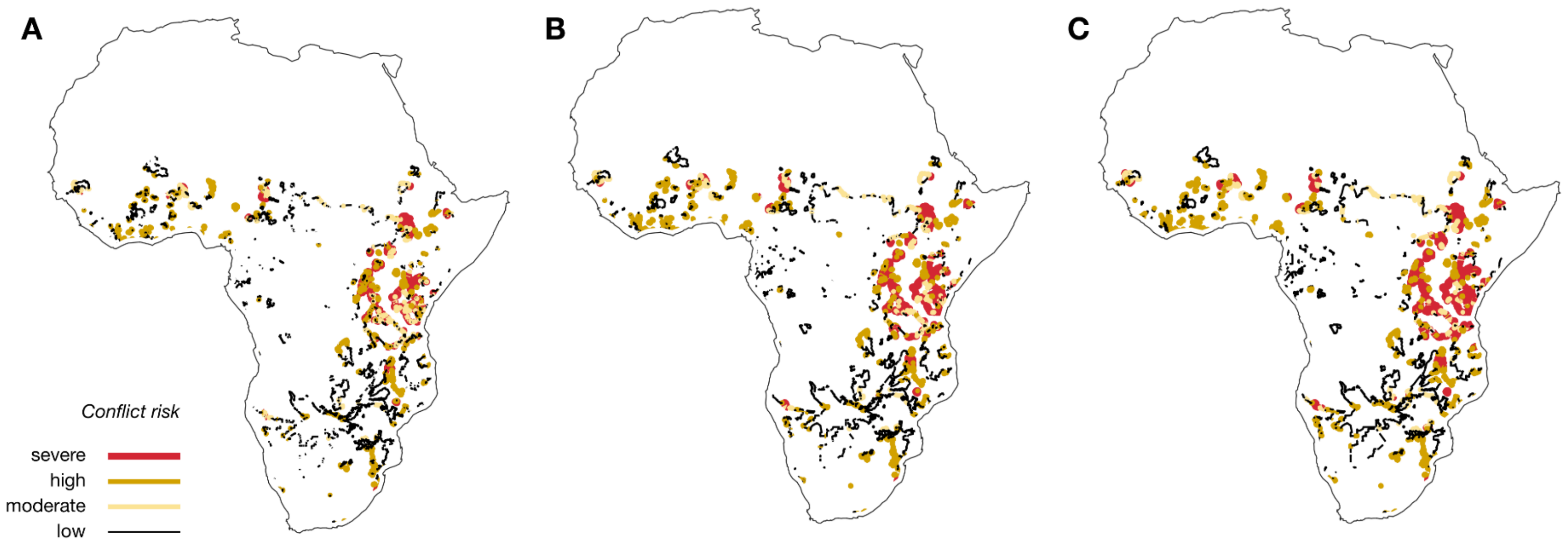

Supplementary Figure 6. Less certain 100-m segments in the identified areas at risk of conflict on the perimeter of the extended ranges of African lions (*Panthera leo*) and African elephants (*Loxodonta africana* and *Loxodonta cyclotis*) at (A) 10, (B) 20, and (C) 30-km buffer distances. Specifically, Latin hypercube sampling was used to randomly vary the minimum distance values between the extended ranges and human pressure maps 100 times across ±10% of the values. The resulting 100 randomly created distance values were then averaged and the 100-m segments falling outside of the analyzed buffer distances of 10, 20, and 30 km identified as less certain.

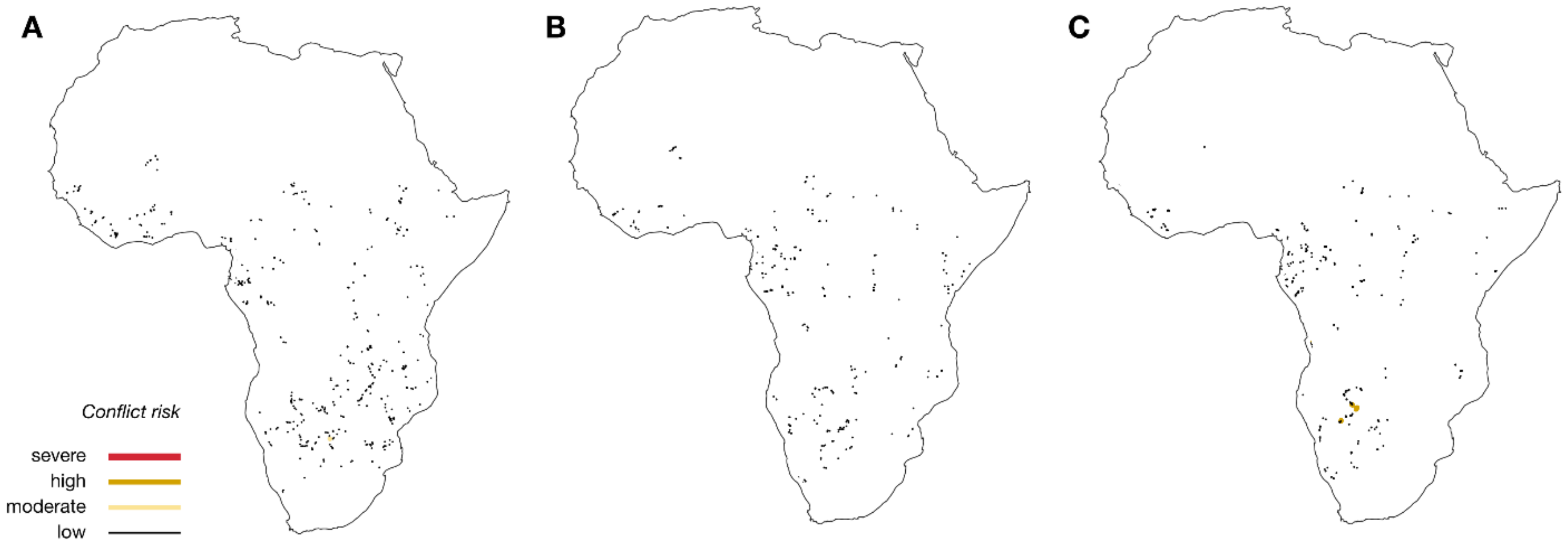

Supplementary Figure 7. Certainty of African lions (*Panthera leo*) and African elephants (*Loxodonta africana* and *Loxodonta cyclotis*) presence in areas at severe risk of conflict. Certainty is considered higher where species ranges and protected areas overlap. Certainty is considered lower where species ranges and protected areas do not overlap.

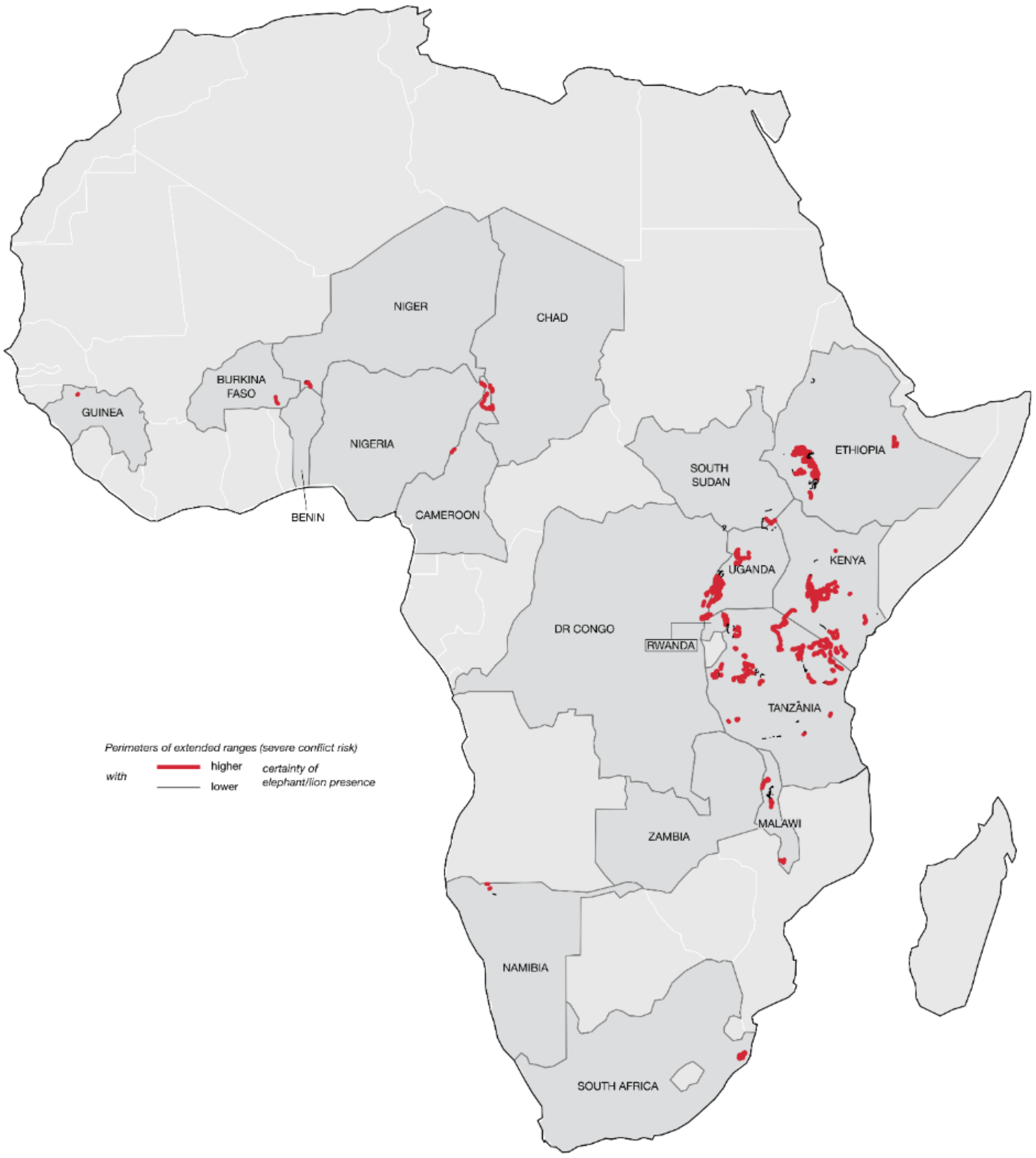

Supplementary Figure 8. Boxplot of the equivalent annual annuity (EAA) of building and maintaining mitigation fences in areas at severe risk of conflict between humans and African lions (*Panthera leo*) and African elephants (*Loxodonta africana* and *Loxodonta cyclotis*). Different panels are for 10- (A), 20- (B), and 30- (C) km buffer distances. Whisker represent range from minimum to maximum, box indicates 25 and 75 percentile, and horizontal line represents median. Plotted dots represent 100 EAA values calculated by varying all economic model parameters randomly across ±10% of the values of each parameter. Dots outside the whisker boundaries are outliers.

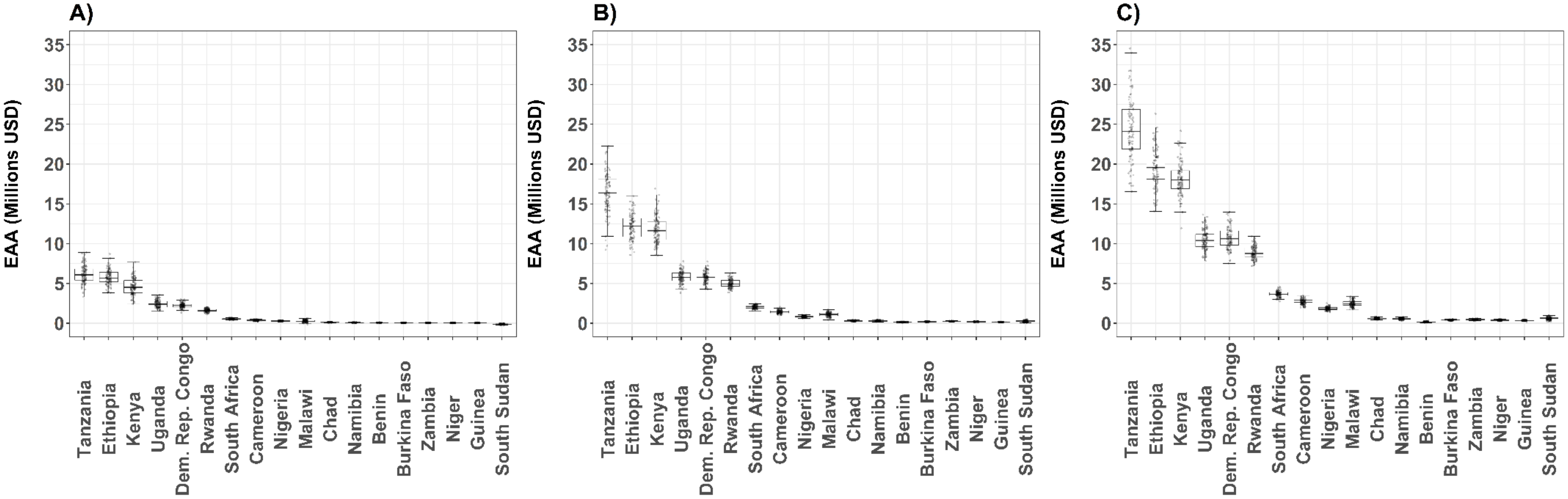

Supplementary Figure 9. Boxplot of the equivalent annual annuity (EAA) of building and maintaining mitigation fences in areas at high risk of conflict between humans and African lions (*Panthera leo*) and African elephants (*Loxodonta africana* and *Loxodonta cyclotis*). Whisker represent range from minimum to maximum, box indicates 25 and 75 percentile, and horizontal line represents median. Plotted dots represent 100 EAA values calculated by varying all economic model parameters randomly across ±10% of the values of each parameter. Dots outside the whisker boundaries are outliers. See Figure 3 in the main text for a comparison with countries at severe risk of conflict.

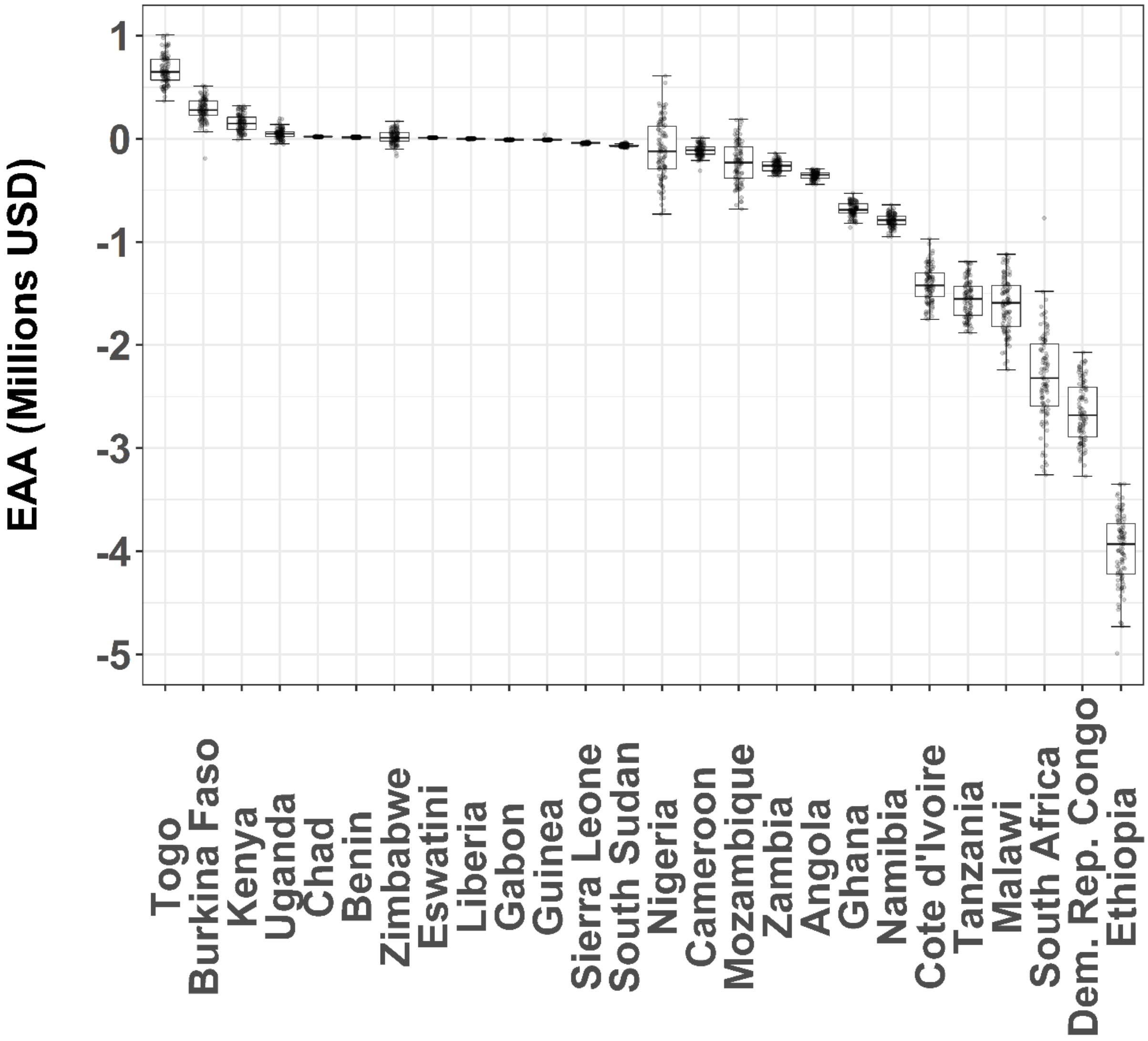

Supplementary Tables

Supplementary Table 1. List or variables used in the generalized linear mixed models. Cattle density and proportion of crops were used for lions and elephants respectively.

Variable	Explanation	Source
Human population density in buffer	Human population density in a buffer of 10 km from each conservation site based on Gridded Population of the World data.	https://sedac.ciesin.columbia.edu/data/collection/gpw-v4
Cattle density in buffer	Cattle density in a buffer of 10 km from each conservation site based on the Gridded Livestock of the World data.	https://dataverse.harvard.edu/dataverse/glw
Proportion of crops in buffer	Proportion of croplands in a buffer of 10 km from each conservation site based on the Global Land Cover map.	https://land.copernicus.eu/global/products/lc
Gross Domestic Product	Each country's gross domestic product (GDP) per capita (corrected for purchasing-power parity) for 2018 as an index of total wealth from the World Bank.	https://data.worldbank.org/indicator/ny.gdp.pcap.cd
Conservation funding	Conservation expenditure for each country	Waldron et al. [1]
Ibrahim Index of African Governance	African specific indicator of governance for 2017, which includes safety and rule of law, participation and human rights, sustainable economic opportunity, and human development indicators in its normalized overall score.	http://iiag.online/

Supplementary Table 2. Contribution of range countries to African lion (*Panthera leo*) and African elephant (*Loxodonta africana* and *Loxodonta cyclotis*) conservation in Africa.

Region	Country	Perimeter elephant range (km)	Perimeter lion range (km)
Central	Cameroon	5086	1654
Central	Central African Republic	1792	1434
Central	Chad	3976	1627
Central	Congo	5393	0
Central	Democratic Republic of Congo	11199	5144
Central	Equatorial Guinea	181	0
Central	Gabon	4732	0
Eastern	Eritrea	451	0
Eastern	Ethiopia	4924	7476
Eastern	Kenya	6768	5771
Eastern	Rwanda	299	299
Eastern	Somalia	511	1060
Eastern	South Sudan	2662	2595
Eastern	Sudan	884	884
Eastern	Tanzania	10697	10426
Eastern	Uganda	2904	2346
Southern	Angola	2384	2313
Southern	Botswana	3391	3375
Southern	Eswatini	287	0
Southern	Malawi	2090	1516
Southern	Mozambique	7697	7473
Southern	Namibia	6455	6192
Southern	South Africa	5326	1878
Southern	Zambia	7536	6188
Southern	Zimbabwe	6624	5796
West	Benin	649	649
West	Burkina Faso	2399	444
West	Côte d'Ivoire	5105	0
West	Ghana	2802	0
West	Guinea	830	444
West	Guinea-Bissau	373	0
West	Liberia	1736	0
West	Mali	1378	244
West	Niger	315	190
West	Nigeria	2882	770
West	Senegal	763	763
West	Sierra Leone	556	0
West	Togo	718	0

Supplementary Table 3. Top-ranked generalized linear models testing five potential predictors of African lion (*Panthera leo*) (a) and African elephant (*Loxodonta africana and Loxodonta cyclotis*) (b) population numbers across conservation sites in Africa. The 10-fold cross-validation, top-ranked models had a mean prediction error of 9% for lion and 11% for elephant. Low percentage of deviance explained suggests that other factors beyond those evaluated here also influence population numbers.

**Model**	**Log-likelihood**	**Bayesian information criterion weight**	**% of deviance explained**
(a) **Lion**			
pop ~ 1 + hum_pop_dens + funding_cons	395.79	0.61	28
pop ~ 1 + cattle_density + hum_pop_dens + funding_cons	399.53	0.09	29
pop ~ 1 + hum_pop_dens + funding_cons + governance	399.81	0.08	29
pop ~ 1 + hum_pop_dens + funding_cons + GDP	399.91	0.08	28
pop ~ 1 + hum_pop_dens	400.11	0.07	18
pop ~ 1 + hum_pop_dens + GDP	400.49	0.07	22
(b) **Elephant**			
pop ~ 1 + hum_pop_dens + GDP	3463.58	0.54	31
pop ~ 1 + hum_pop_dens + crop + GDP	3466.25	0.14	31
pop ~ 1 + hum_pop_dens + crop + GDP + governance	3466.24	0.14	31
pop ~ 1 + hum_pop_dens + funding_cons + GDP + governance	3467.86	0.06	40
pop ~ 1 + hum_pop_dens + funding_cons + GDP	3467.85	0.06	41
pop ~ 1 + hum_pop_dens + GDP + governance	3468.44	0.06	30

hum_pop_dens = human population density; funding_cons = conservation expenditure; cattle_density = cattle density; governance = Ibrahim Index of African Governance; GDP = gross domestic product per capita; crop = proportion of crops.

Supplementary Table 4. Countries and lengths of perimeters of African lion (*Panthera leo*) and African elephant (*Loxodonta africana* and *Loxodonta cyclotis*) ranges at severe risk of conflict at 10- (A), 20- (B), and 30- (C) km buffer distances.

Country	10 km	20 km	30 km
Tanzania	3073	4854	6548
Kenya	2592	3329	3917
Ethiopia	2138	2614	2982
Uganda	1526	1886	2042
Malawi	485	727	831
Cameroon	300	490	591
South Sudan	297	385	497
Rwanda	295	299	299
DR Congo	265	483	741
South Africa	142	173	210
Niger	65	90	108
Chad	56	95	147
Namibia	45	133	206
Burkina Faso	31	86	142
Benin	24	93	199
Nigeria	22	65	99
Zambia	14	30	115
Guinea	8	52	185
Zimbabwe		3	108
Senegal		1	84
Somalia			38
Mozambique			11

Supplementary Table 5. Percentage of segments used in the global sensitivity analysis that are uncertain to the geographic location of human pressures at 10-, 20-, and 30-km buffer distances.

		**% uncertain**	
	**10 km**	**20 km**	**30 km**
Severe	0	0	0
High	0	0	0.16
Moderate	0	0	0
Low	0.09	0.07	0.20

Supplementary Table 6. Perimeter length-to-area ratio of the ranges of migratory mammals in orders Cetartiodactyla, Perissodactyla, Primates, and Carnivora whose ranges were identified as intersecting severe-risk conflict areas. Shaded in grey are species for which the ratio increases with fencing; for other species, the fencing had no material effect on their ranging.

		Without fences			With fences		
**Species**	**IUCN Category**	**Area (km^2)**	**Perimeter (km)**	**Ratio**	**Area (km^2)**	**Perimeter (km)**	**Ratio**
African buffalo (*Syncerus caffer*)	Near threatened	7384504	465679	0.06	7384363	472857	0.06
African wild dog (*Lycaon pictus*)	Endangered	1238060	45796	0.04	1238030	46908	0.04
Cheetah (*Acinonyx jubatus*)	Vulnerable	2891128	83188	0.03	2891090	85010	0.03
Chimpanzee (*Pan troglodytes*)	Endangered	2188014	48987	0.02	2188000	49662	0.02
Common wildebeest (*Connochaetes*	Least concern	2875749	204610	0.07	2875721	205965	0.07
Eland (*Tragelaphus oryx*)	Least concern	6040980	526294	0.09	6040880	531489	0.09
Gerenuk (*Litocranius walleri*)	Near threatened	1368340	35579	0.03	1368320	36721	0.03
Giraffe (*Giraffa camelopardalis*)	Vulnerable	1769505	194865	0.11	1769427	198724	0.11
Greater kudu (*Tragelaphus strepsiceros*)	Least concern	5307290	426226	0.08	5307200	430758	0.08
Grevy's zebra (*Equus grevyi*)	Endangered	92164	5491	0.06	92152	6075	0.07
Impala (*Aepyceros melampus*)	Least concern	2857720	308068	0.11	2857610	313394	0.11
Kob (*Kobus kob*)	Least concern	2946559	469911	0.16	2946490	473132	0.16
Leopard (*Panthera pardus*)	Vulnerable	6225690	281694	0.05	6225590	286582	0.05
Lesser kudu (*Tragelaphus imberbis*)	Near threatened	1372650	59886	0.04	1372620	61704.2	0.04
Plains zebra (*Equus quagga*)	Near threatened	2020350	224845	0.11	2020250	229486	0.11
Red-fronted gazelle (Eudorcas rufifrons)	Vulnerable	1885050	156339	0.08	1885040	156675	0.08
Roan antelope (*Hippotragus equinus*)	Least concern	4883669	434269	0.09	4883618	436457	0.09
Sable antelope (*Hippotragus niger*)	Least concern	2678277	286201	0.11	2678246	287762	0.11
Thomson's gazelle (*Eudorcas thomsonii*)	Least concern	105191	3517	0.03	105186	3735	0.04
Topi (*Damaliscus lunatus*)	Least concern	1733393	103916	0.06	1733373	104882	0.06

Supplementary Table 7. Projected human population density (people/km^2). Countries with areas at severe risk of conflict are shaded in grey. Data: https://population.un.org/wpp/Download/Probabilistic/Population/

Country	2025	2050	2100
Angola	31	62	151
Benin	123	215	419
Burkina Faso	88	159	304
Cameroon	64	107	191
Chad	15	27	49
Côte d'Ivoire	94	161	304
Democratic Republic of Congo	46	86	160
Eswatini	71	99	125
Ethiopia	130	205	294
Gabon	10	15	23
Ghana	151	229	347
Guinea	61	106	184
Kenya	105	161	220
Liberia	59	97	161
Malawi	232	405	706
Mozambique	46	83	157
Namibia	3	5	7
Niger	23	52	130
Nigeria	256	441	805
Rwanda	591	934	1354
Sierra Leone	122	179	231
South Africa	52	62	65
South Sudan	20	32	51
Tanzania	171	283	495
Togo	261	446	682
Uganda	78	146	322
Zambia	29	53	110
Zimbabwe	42	62	80

Supplementary Table 8. Millions of people living within a 10 km distance from areas at severe risk of conflict, and equivalent annual annuity per capita (EAAPC) in USD resulting from building and maintaining mitigation fences in areas at severe risk of conflict between humans and African lions and African elephants.

Country	Millions of people	EAAPC
Kenya	4.99	0.93 (±0.19)
Tanzania	3.84	1.56 (±0.30)
Uganda	3.23	0.78 (±0.16)
Ethiopia	3.04	1.89 (±0.30)
Cameroon	1.87	0.19 (±0.04)
Dem. Rep. Congo	1.50	1.50 (±0.18)
Rwanda	1.46	1.11 (±0.13)
Chad	1.39	0.08 (±0.02)
Malawi	0.58	0.44 (±0.20)
South Sudan	0.43	-0.25 (±0.15)
South Africa	0.15	3.59 (±0.44)
Nigeria	0.09	3.19 (±0.37)
Niger	0.05	0.81 (±0.30)
Namibia	0.03	3.15 (±0.70)
Burkina Faso	0.03	2.32 (±0.33)
Zambia	0.02	3.29 (±0.39)
Benin	0.01	8.56 (±1.21)
Guinea	0.01	3.17 (±0.50)

Supplementary Table 9. Country specific discount rates used as part of the economic analyses. *The central bank policy rate is used in some countries as the discount rate. Sources: International Monetary Fund (IMF): https://data.imf.org/regular.aspx?key=61545855; African Development Bank (AFDB): https://knoema.com/AFSED2020/afdb-socio-economic-database-1960-2021; Central Bank of Liberia (CBL): https://www.cbl.org.lr/doc/2016AnnualReport.pdf.; Reserve Bank of Malawi (RBM): https://www.rbm.mw/statistics/bankrates/

**Country**	**Rate**	**Year**	**Source**
Angola	20	2017	IMF
Benin	2. 5*	2017	IMF
Burkina Faso	2. 5*	2017	IMF
Cameroon	2.95	2017	IMF
Chad	2.95	2017	IMF
Cote d'Ivoire	2. 5*	2017	IMF
Democratic Republic of the Congo	21	2017	IMF
Eswatini	7.25	2017	IMF
Ethiopia	12	1995	AFDB
Gabon	2.95	2017	IMF
Ghana	20*	2017	IMF
Guinea	5	2017	IMF
Kenya	10*	2017	IMF
Liberia	3.2	2016	CBL
Malawi	12*	2020	RBM
Mozambique	9.95	2017	IMF
Namibia	7	2009	AFDB
Niger	2.5*	2017	IMF
Nigeria	14*	2017	IMF
Rwanda	9.5	2017	IMF
Sierra Leone	14.5*	2017	IMF
South Africa	6.75*	2017	IMF
South Sudan	5	2017	IMF
Tanzania	9	2017	IMF
Togo	2.5*	2017	IMF
Uganda	15	2015	IMF
Zambia	11.46	2017	IMF
Zimbabwe	6.91	2017	IMF

References

1. Waldron, A. et al. Targeting global conservation funding to limit immediate biodiversity declines. *Proc. Natl Acad. Sci. USA* **110**, 12144-12148 (2013).
