# Supplementary material for: A pan-African spatial assessment of human conflicts with lions and elephants
Source: Nat Commun. 2021 May 20;12:2978. doi: 10.1038/s41467-021-23283-w (PMC8138028; doi:10.1038/s41467-021-23283-w)
Supplement: Supplementary file 2 — Reporting Summary [file 41467_2021_23283_MOESM2_ESM.pdf]

nature research

Corresponding author(s): Enrico Di Minin

Last updated by author(s): Mar 29, 2021

Reporting Summary

Nature Research wishes to improve the reproducibility of the work that we publish. This form provides structure for consistency and transparency in reporting. For further information on Nature Research policies, see our Editorial Policies and the Editorial Policy Checklist.

Statistics

For all statistical analyses, confirm that the following items are present in the figure legend, table legend, main text, or Methods section.

n/a	Confirmed	
☒	☐	The exact sample size (*n*) for each experimental group/condition, given as a discrete number and unit of measurement
☒	☐	A statement on whether measurements were taken from distinct samples or whether the same sample was measured repeatedly
☒	☐	The statistical test(s) used AND whether they are one- or two-sided<br>*Only common tests should be described solely by name; describe more complex techniques in the Methods section.*
☐	☒	A description of all covariates tested
☒	☐	A description of any assumptions or corrections, such as tests of normality and adjustment for multiple comparisons
☐	☒	A full description of the statistical parameters including central tendency (e.g. means) or other basic estimates (e.g. regression coefficient) AND variation (e.g. standard deviation) or associated estimates of uncertainty (e.g. confidence intervals)
☒	☐	For null hypothesis testing, the test statistic (e.g. *F*, *t*, *r*) with confidence intervals, effect sizes, degrees of freedom and *P* value noted<br>*Give P values as exact values whenever suitable.*
☒	☐	For Bayesian analysis, information on the choice of priors and Markov chain Monte Carlo settings
☒	☐	For hierarchical and complex designs, identification of the appropriate level for tests and full reporting of outcomes
☒	☐	Estimates of effect sizes (e.g. Cohen's *d*, Pearson's *r*), indicating how they were calculated

Our web collection on statistics for biologists contains articles on many of the points above.

Software and code

Policy information about availability of computer code

Data collection	Data preprocessing was carried out using the open source database PostgreSQL 11.4 (https://www.postgresql.org/about/) with the GIS extensions of PostGIS 2.5 (https://postgis.net/) and Python v. 3.7.0 .
Data analysis	Conflict mapping and range fragmentation analyses used PostgreSQL 11.4 and PostGIS 2.5 and Python v. 3.7.0; statistical and economic analyses used R v. 3.6.0; sensitivity analyses used PostgreSQL 11.4 and PostGIS 2.5 and Python v. 3.7.0 and R v. 3.6.0. We determined the magnitude and direction of the coefficients for the independent variables with multi-model averaging implemented in the R package glmulti.

For manuscripts utilizing custom algorithms or software that are central to the research but not yet described in published literature, software must be made available to editors and reviewers. We strongly encourage code deposition in a community repository (e.g. GitHub). See the Nature Research guidelines for submitting code & software for further information.

Data

Policy information about availability of data

All manuscripts must include a data availability statement. This statement should provide the following information, where applicable:
- Accession codes, unique identifiers, or web links for publicly available datasets
- A list of figures that have associated raw data
- A description of any restrictions on data availability

Information on the distribution and population sizes of lion and elephant are available from the IUCN Cat and African Elephant Specialist Groups. The study used openly available datasets of Gridded Population of the World Version 4, Gridded Livestock of the World database and crop maps available from the Copernicus Global Land Cover map with references provided in the Methods section. Range maps for all terrestrial mammal species used in the fragmentation analyses are available from the IUCN Red List portal (www.iucnredlist.org/). The data on protected areas were available from the World Database on Protected Areas (http://www.protectedplanet.net). Data for the economic analyses are openly available from sources such as FAO and links are provided in the Methods section. Our

conflict risk maps are available to download from: https://etsin.fairdata.fi/dataset/d0ac647a-4d73-4117-89de-9d194215f948

Field-specific reporting

Please select the one below that is the best fit for your research. If you are not sure, read the appropriate sections before making your selection.

☐ Life sciences ☐ Behavioural & social sciences ☒ Ecological, evolutionary & environmental sciences

For a reference copy of the document with all sections, see nature.com/documents/nr-reporting-summary-flat.pdf

Ecological, evolutionary & environmental sciences study design

All studies must disclose on these points even when the disclosure is negative.

Study description	We mapped spatial conflict between lions and elephants and humans across Africa, by combining information on the distribution of lions and elephants with spatial information on human population density, cropland, and cattle density, as these are considered the most important drivers of human-wildlife conflict in Africa. By focusing on the areas at severe risk of conflict, we estimated the return on investment of building and maintaining mitigation fences in these areas and considered whether proposed fence-lines would affect other migratory mammals.
Research sample	Information on the distribution and population sizes of lion and elephant are available from the IUCN Cat and African Elephant Specialist Groups. The study used openly available datasets of Gridded Population of the World Version 4, Gridded Livestock of the World database and crop maps available from the Copernicus Global Land Cover map with references provided in the Methods section. Range maps for all terrestrial mammal species used in the fragmentation analyses are available from the IUCN Red List portal (www.iucnredlist.org/). The data on protected areas were available from the World Database on Protected Areas (http://www.protectedplanet.net). Data for the economic analyses are openly available from sources such as FAO and links are provided in the Methods section.
Sampling strategy	We carried out multiple sensitivity analyses to account for uncertainty in the distribution of elephants and lions and the human pressure layers and in model estimates when calculating the equivalent annual annuity of building and maintaining mitigation fences.
Data collection	Data preprocessing was carried out using the open source database PostgreSQL 11.4 (https://www.postgresql.org/about/) with the GIS extensions of PostGIS 2.5 (https://postgis.net/) and Python v. 3.7.0 .
Timing and spatial scale	This is an Africa-wide analysis combining several datasets. Data on the number of African lions and elephants was available for 2018 and 2016 respectively. Human population data are for 2020; cropland data are for 2015; and cattle data are for 2010. Economic data are for 2017.
Data exclusions	No data were excluded from the analyses.
Reproducibility	We used openly available datasets and refer to the IUCN Cat and African elephant specialist groups for information on the distribution and population sizes of elephants and lions. In addition, we provide conflict risk maps for download.
Randomization	Not applicable. Research did not involve any experiments where study participants were randomly chosen.
Blinding	Not applicable. Data aquisition and processing did not involve any data blinding as no study participants were involved.

Did the study involve field work? ☐ Yes ☒ No

Reporting for specific materials, systems and methods

We require information from authors about some types of materials, experimental systems and methods used in many studies. Here, indicate whether each material, system or method listed is relevant to your study. If you are not sure if a list item applies to your research, read the appropriate section before selecting a response.

Materials & experimental systems

n/a	Involved in the study	
☒	☐	Antibodies
☒	☐	Eukaryotic cell lines
☒	☐	Palaeontology and archaeology
☒	☐	Animals and other organisms
☒	☐	Human research participants
☒	☐	Clinical data
☒	☐	Dual use research of concern

Methods

n/a	Involved in the study	
☒	☐	ChIP-seq
☒	☐	Flow cytometry
☒	☐	MRI-based neuroimaging
